# The systematic development of guidance for parents on talking to children of primary school age about weight

**DOI:** 10.1186/s12889-023-16527-5

**Published:** 2023-09-04

**Authors:** Fiona B. Gillison, Elisabeth B. Grey, Fran Baber, Angel Chater, Lou Atkinson, Alison Gahagan

**Affiliations:** 1 Centre for Motivation and Health Behaviour Change, Department for Health, University of Bath, Bath, UK; 2 Population Health Sciences, Bristol Medical School, University of Bristol, Bristol, UK; 3 Institute for Sport and Physical Activity Research (ISPAR), Centre for Health, Wellbeing and Behaviour Change, University of Bedfordshire, Polhill Avenue, Bedford, MK41 9EA UK; 4 Centre for Behaviour Change, University College London, 1-19 Torrington Place, London, WC1E 7HB UK; 5 University of Warwick, Coventry, CV4 7AL UK; 6 Department of Health and Social Care, Office for Health Improvement and Disparities, 39 Victoria Street, London, SW1H 0EU UK

**Keywords:** Childhood obesity, Health communication, Weight talk, Guidance development, Parent communication

## Abstract

**Background:**

The prevalence of overweight and obesity in children is increasing, alongside increases in rates of children’s anxiety and worry about their weight. In many countries children’s weight is measured, and parents are made aware if a child has been classified as having overweight or obesity. However, many parents are concerned that raising their child’s awareness of their weight, and talking to children about their weight could harm their wellbeing. The aim of this project was to develop guidance for parents on constructive ways to talk to children of primary school age about weight when they need to.

**Methods:**

The project followed a mixed-methods design: Phase 1 involved the collation of evidence including (a) two previously published systematic literature reviews to identify the associations between parent–child weight talk, and broader health discussions, and children’s wellbeing, (b) interviews with children, and (c) development and refinement of narrative messaging (previously published). In Phase 2 we developed a conceptual model and mapped primary findings to techniques and content within a draft guidance document for parents on talking to children about weight. Phase 3 involved a modified Delphi process with 29 stakeholders to refine and agree a final version.

**Results:**

An acceptable draft guidance was agreed following two stages of feedback from Delphi participants. Key areas for debate and adaptation included: encouraging discussion about health and growth with lesser focus on weight; finding ways to reduce stigma and perceptions of blame; emphasising a whole-family approach; inclusive representation of diversity among children and families.

**Conclusions:**

Consensus on the content of guidance for parents on talking to children about weight was achieved through a process of evidence review and stakeholder and expert engagement. The next steps are to measure the impact of the resource on improving the experience of parents and children in communicating about weight.

**Supplementary Information:**

The online version contains supplementary material available at 10.1186/s12889-023-16527-5.

## Introduction

Childhood obesity is a significant global public health concern [1–3]. Children classified as living with obesity or overweight are at increased risk of obesity in adulthood, and the subsequent health and social risks that this incurs [4]. Consequently, many countries have implemented policies to monitor levels of childhood obesity, to provide a baseline against which the need for intervention strategies can be justified and initiatives to reduce obesity prevalence can be evaluated [5]. The National Child Measurement Programme (NCMP) in England is one such programme, introduced in 2006 to weigh and measure all children in Reception (aged 4–5) and Year 6 (age 10–11) in English schools whose parents have not opted out [6]. Prior to the COVID-19 pandemic, the NCMP reached over 95% of eligible children and thus provides comprehensive estimates of childhood obesity since the programme started. Similar cohort-wide measurements are made in schools throughout Europe through the WHO COSI surveillance initiative [7].

While the NCMP and other similar schemes were originally set up as surveillance tools, many areas now provide feedback on the weight status of children who are outside a healthy weight range to their primary carer [8]. In part this is to ensure parents are aware of their child’s weight status and inferred health risk so that they are able to make informed decisions about whether or not to make support changes to children’s physical activity and diet, given that up to 51% of parents underestimate the weight status of a child with overweight or obesity [9]. Parents receiving feedback that their child is overweight are usually provided with sources of further support, which that could be online (e.g., Better health, healthier families [10]) or local child weight management programmes [8]. However, providing this feedback and raising parental awareness about a child’s weight can lead to angry and upset responses from some parents [11–13], and has been criticised due to the association between parents’ and children’s perceptions of being overweight, and poorer mental health outcomes in children [14, 15]. While no specific association between such measurement programmes and the development of children’s weight dissatisfaction has been identified, weight control behaviours among children and adolescents have increased over recent years [16, 17]. Parents report fears that the measurement and feedback process may trigger unplanned conversations with their child about weight that would not otherwise have happened, which may be sufficient to start a sequence of events that negatively impact their child’s wellbeing [11].

A systematic review [18] of studies exploring the association between parents talking to children about their weight, and children’s mental health and wellbeing, found a positive association in cross-sectional studies (i.e., more communication was associated with poorer mental health). However, the relationships investigated were not causal, and as different forms of communication co-occurred it was not possible to separate the effects of interactions that might be expected to have negative effects (e.g., teasing and weight criticism), from interactions that parents intended to be more supportive, even if emphasising a child’s responsibility (e.g., encouragement for children to change their behaviour in order to control their weight). The four intervention studies within the review [18] along with other research [19] indicate that constructive ways of talking to children about weight, particularly by shifting emphasis from weight to health behaviours, may actually improve children’s wellbeing. Similarly, most studies investigating the direct effects of measurement and feedback (as opposed to broader societal and cultural language and changes) on negative outcomes for children do not show a clear negative effect [20, 21]. Nevertheless, the finding that some forms of communication may have a negative impact on children’s wellbeing indicates that we have a responsibility to ensure that this evidence is shared and put into practice. That is, we should ensure that if policies or interactions with health professionals may prompt conversations about weight between parents and children, parents are provided with guidance on how to communicate in a way that is most likely to result in positive outcomes.

Public Health England (PHE), now the Office for Health Improvement and Disparities (OHID), issued guidance to health care professionals (HCPs) in 2019 on how to talk to parents positively about children’s weight when a child is identified as having overweight or obesity [22], but there is no equivalent guidance for parents on talking to their children. Therefore, the aim of the project reported in this paper was to conduct a collaborative programme of work to identify evidence-based best practice for parents on how to talk to children about their weight in ways that positively support their wellbeing and avoid exacerbating weight dissatisfaction, and to translate this into a usable guidance tool. The guidance was designed to accompany the NCMP in the first instance but is applicable for all parents of primary school aged children in other settings where the same concerns are raised, for example in children’s health check-ups or when children themselves raise concerns to parents. This paper provides a real-world example of the strategic and rigorous process taken to produce a guidance tool where there is no single source of evidence, or single correct answer on which to base best practice.

## Methods

We took a mixed-methods, three-phase approach to development of the guidance. Phase 1 involved the collection and collation of research evidence around the impact of different types of weight and health communication between parents and children on children’s wellbeing. Where evidence was not available, we addressed these gaps through primary research, stakeholder engagement activities, or drawing on evidence in related settings [23]. Phase 2 involved translating the key points from the evidence review into specific content of a guidance document. Phase 3 involved a modified Delphi process to refine and agree a final version of a guidance document for implementation. This final phase facilitated the inevitable compromises needed to navigate between academic evidence, clinical practice expertise, acceptability to stakeholders, and the practicalities of producing a readable and usable resource for parents. For parsimony, throughout the paper we use the term ‘parent’ to indicate either a parent or primary carer.

### Phase 1: Collection and collation of evidence

#### Systematic literature review

A systematic review (published elsewhere, [18]) was initially conducted by searching eight databases using the search terms; (child* OR *daughter* OR son OR adolescent OR youth OR teen* OR young) AND (*parent* OR *mother* OR *father OR caregiver*) AND (weight talk OR communication OR body image OR eating disorder OR dysfunctional eating OR wellbeing) AND (weight OR obes* OR overweight) to analyse the association between parent–child weight-talk and child wellbeing. Well-being was defined broadly to include positive and negative mood states, mental health and ill-health (including dysfunctional eating), and body dissatisfaction, and all types of parent–child communication were included for children of all ages up to 18. Quantitative studies reporting on associations between parent communication and children’s wellbeing, or on the outcomes of interventions were included; qualitative designs were excluded. Full methods and findings have been previously published [18], however in brief, 38 studies reported on associative outcomes that provided useful insights, but only four were interventions. Of these, only one isolated parent–child communication from other aspects of weight management approaches.

A second, broader review (published elsewhere, [23]) was subsequently conducted to synthesise research on the causal links between communication between parents and children (aged 4–12) in this and other health domains where stigma or concern for wellbeing may deter parents from initiating a discussion. This latter review was published in 2022 [23] and drew on data published in five databases up to April 2020. The search strategy was inclusive of intervention studies (if they provided guidance, support or training to parents on how to talk to children about their health and health behaviours), observational studies, qualitative and quantitative designs and analysis of secondary data. This search strategy would have identified any more recent studies meeting the inclusion criteria of the earlier review [18], in addition to a wider set of studies on other sensitive health topics. The end-date for study eligibility reflects the latest point at which we could include new data into the materials collated to start creating the guidance.

#### Review of existing guidance

To ensure that new guidance was necessary and would build on current good practice, we searched for any existing guidance through a grey literature review of documents available between October 2019 and April 2020, and updated in September 2020, using the Google search engine. The inclusion criteria were for guidance available in English, aimed explicitly at parents about communication with children about body weight or size. Broad key terms (e.g., ‘child weight talk’) and questions (e.g., ‘how do I talk to my child about weight?’) were used to run searches, and retrievals from the first five pages of results were screened, including informal sources such as blog posts and newspaper or magazine articles. Professionals working in public health and child psychology identified from the authors’ professional networks were also contacted to advise of any known resources. The *a priori* criteria for determining the need for new guidance are set out in Table 1.

**Table 1:** Criteria for determining the need for new guidance

Requirement	Rationale
Absence of guidance developed or adapted and tested in the UK	- to maximise acceptability and relevance to parents living in the UK
Absence of guidance with a clear research evidence base	- to be in line with requirements for evidence-based practice in public health - to maximise acceptability to HCPs, and trustworthiness to all stakeholders
Absence of comprehensive content, including guidance for parents on (i) deciding whether or not to talk to children about weight (ii) raising the topic of weight (iii) continuing conversations about weight	- to address expressed needs of parents, identified through past research and PPIE - to address expressed needs of HCPs in terms of the questions parents ask of them
Absence of guidance reflecting a biopsychosocial model of obesity (i.e., acknowledging environmental and social determinants of obesity)	- to align with current public health focus on the importance of a systems-wide approach to obesity prevention [24]

*HCP* Health Care Professional, *PPIE* Patient and Public Involvement and Engagement

Fourteen guidance documents were identified (Additional File 1) which had been developed/updated from 2010 to 2019 (where noted). Not all were comprehensive (e.g., providing guidance solely on whether or not to talk to children about weight, rather than how to go about it). Seven were produced in the USA, three in the UK, two in Australia, one in Ireland and one provided for Europe in general. The two most comprehensive resources appeared to have a similar aim to the current research: ‘Weigh In’ from the US [25], and ‘Confident Body, Confident Child’ (CBCC) developed in Australia [26]. Both sets of guidance had been developed with experts from academia and clinical practice, are available to parents on interactive websites, and make reference to sources of evidence (although all references cited for both resources were over a decade old when this review was conducted). CBCC has also been tested as part of a workshop intervention [19, 27]. In the UK, brief guidance developed with experts from academia and clinical practice in the mid-2000s was available through the Weight Concern charity website at the time the review was conducted, but the charity is now no longer in operation. As none of the guidance documents met all of our a priori criteria, we progressed with the development of new evidence-based guidance.

#### Interviews with children on their experience and expectations of weight monitoring

Although much has been published on parents’ views of weight measurement programmes, such as the NCMP [11–13, 21], and of the challenges health practitioners experience when talking to parents about children’s weight, the voices of children are largely absent from published research. Similarly, little is known about what children who are living with overweight and obesity expect and want from parents; our systematic review search terms were designed to identify studies reporting on this if available [18, 23]. To ensure children’s perspectives were represented we carried out an engagement activity with children, through 11 interviews with children aged 9–11 years (7 female, 4 male) to explore their expectations and understanding of weight measurements in a hypothetical scenario. This age group was chosen due to their proximity to the age and state at which the Year 6 NCMP takes place, and as by this age children have sufficient cognitive development to engage in the hypothetical scenarios included in the interview topic guide. Participants were recruited through a convenience sampling technique, following advertisements in local media and online fora. As the guidance is intended to be suitable for all regardless of the child’s weight, and as an explicit aim to avoid parents having to identify their child as overweight in order to take part, families of children of all body weights were eligible and the interviewed child’s weight status was not requested. In line with ethical approvals, parents were provided with a written information sheet and asked to provide informed consent ahead of participation. Children were provided with an age appropriate information sheet, and the researcher checked through each aspect of participation with them and their parent at the start of the video call, before asking them to formally confirm assent.

Illustrated story cards were used to guide the interview (see Additional File 2), depicting a scenario in which a child took part in a weight measurement programme at school and their parents received a feedback letter informing them that the child was overweight. The researcher read a story card aloud then asked the child questions to explore their thoughts and feelings on each part of the story, before moving on to the next card. Participants’ parents/caregivers were present with the child but were asked not to speak on behalf of, or to prompt their child. All interviews took place remotely via Microsoft Teams or Zoom and were transcribed verbatim; the study was disrupted by the COVID-19 pandemic as the first interview took place in March 2020, but recruitment was then paused until October, ending in November 2020.

**Fig. 1:**
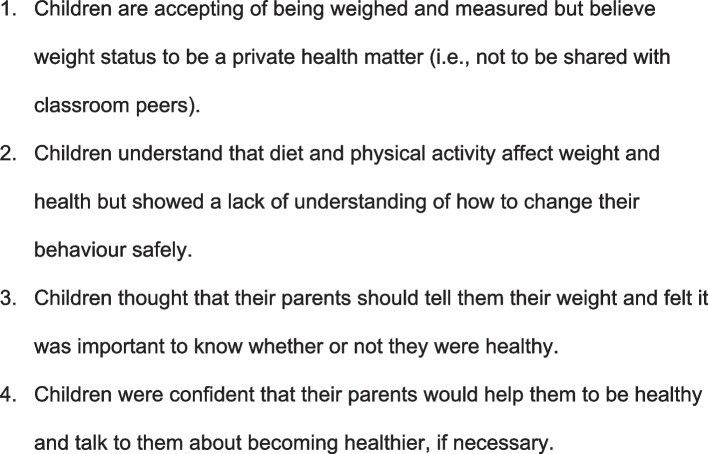
Children’s views about weight and being weighed (*N* = 11; 9–11 years)

The key findings are summarised in Fig. 1, with further detail in supplementary materials (Additional File 2). Similar views were expressed by all children and suggested that children understand that weight is an indicator of health and trust their parents to let them know and take action if their weight is unhealthy.

### Phase 2: Guidance development

#### Conceptual model

**Fig. 2:**
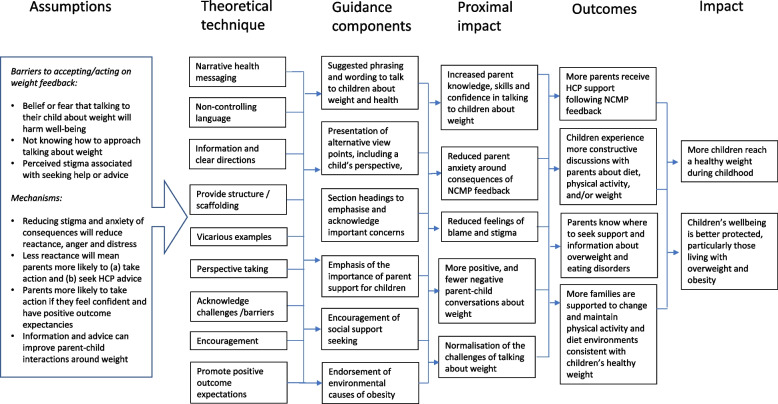
Logic model of guidance effects

The insights from each source of evidence that could inform the content of the guidance document were identified and extracted by FG and EG (Additional File 3). These were translated into either a point of content (e.g., piece of advice) or frame for content (e.g., the spirit, tone or emphasis). A logic model was developed to set out the predicted direct and indirect effects of the guidance through mapping identified parent barriers and needs to specific techniques or content (Fig. 2). Content was included to; reduce stigma, increase confidence in having constructive conversations and deal with arising scenarios, promote social support seeking, normalise challenges in talking about weight with children, and support parents’ autonomy in deciding if and when to have conversations about weight with their child. Immediate benefits were predicted to be on parents’ emotions (e.g., less anxiety, shame or anger), confidence (e.g., in their ability to frame conversations appropriately) and behaviour (e.g., having conversations at the appropriate time, and/or changing the home environment). Indirect benefits for children were predicted to be the receipt of more parental support for dealing with worries about their weight, more support to change their health behaviours to achieve a healthy weight, and experiencing fewer negative conversations about weight (teasing, criticism, blame). Careful considerations were taken to avoid any unintended undesirable outcomes, so none are included in the hypothesised model. However, we note that these should nonetheless be included as possible outcomes in further evaluation (e.g., if the anticipated increase in knowledge and skills does not happen, but more conversations take place as a result of the guidance increasing confidence).

#### Framing the guidance

Psychological reactance is an unpleasant arousal state that occurs as a defence mechanism to feeling labelled within an undesirable and stigmatised group [28]; reactance is evident in parents’ reported feelings of anger, anxiety, guilt and annoyance when receiving feedback that their child does not have a healthy weight status [11, 29, 30]. Reactance results in people rejecting support and acting in the opposite ways to those intended [31]. We drew on theories of health communication, and specifically narrative persuasion [32], to frame the guidance in a way that (a) challenges and reduces stigma and stereotypes of childhood obesity, and (b) reduces barriers to parent engagement stemming from negative psychological reactance. To prevent potential reactance we incorporated narrative messages [33–35] (i.e., case studies and stories) that provide information about health and social issues by describing how a real or fictional character thinks, feels and responds to an unwelcome health message, often reacting negatively at first before ultimately responding positively [36]. Narratives have been reported to be effective in conveying health information to people who are expected to be resistant [34], more effective than didactic messages in changing attitudes to increase support for obesity prevention policies in adults [33, 34, 37] and more effective in making complex information more comprehensible to people with lower educational levels [38].

The narrative accounts included were based on interviews with parents who had previously received feedback from the NCMP informing them that their child was considered to be overweight or very overweight (the term obesity is not used) [39]. Pilot testing of the narrative messages [16] found them to be acceptable and to introduce a novel element to parents by depicting scenarios from the child’s perspective; novelty in messaging is thought to increase readers’ engagement [40].  As a result, throughout the resource we included pull-out quotes depicting children’s perspectives. One new narrative was constructed during the Delphi process (see Phase 3), in response to stakeholder requests for the presentation of a different perspective. This was designed using a similar strategic approach (i.e., specification and inclusion of elements, reference to previous parent interview transcripts), but was not included in the pilot work.

Other techniques to reduce stigma included acknowledging the environmental and external factors that contribute to the development of obesity, and explicitly stating that it is not helpful to blame parents or children for a child’s weight status [33, 41, 42]. We also used person-first language [43] and positive imagery of children (and parents) of varying body sizes and demographics enjoying physical activity and healthy eating, as is consistent with the imagery guidelines of the World Obesity Forum [44].

#### Development of initial draft

**Fig. 3:**
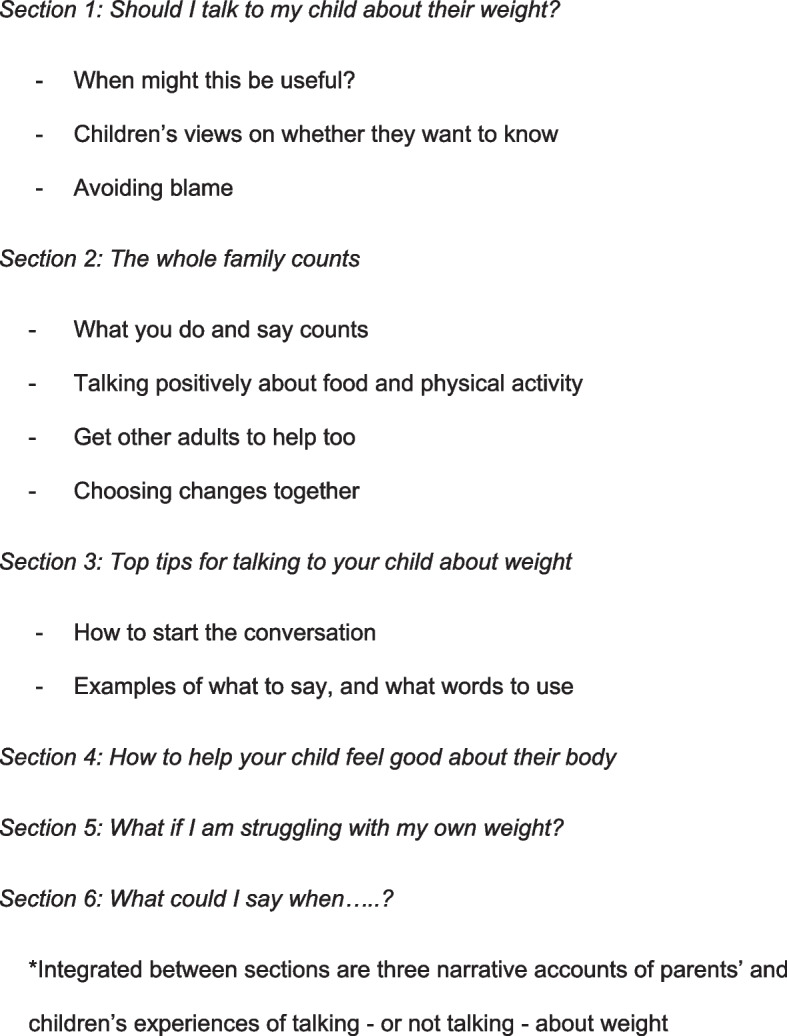
Section headings of the guidance document

An initial draft guidance document was created by FG and EG drawing on all data sources outlined, and integrating them into coherent sections (Fig. 3). This was then refined through an iterative review process with all study authors. The guidance was presented in a PDF format, and to increase the accessibility of the document, text was displayed in short segments or bullet pointed tips and hints, broken up by pull-out quotes and positive images. We intended the guidance to be relevant to all parents concerned about talking about weight with their children, whatever their child’s weight status (e.g., in response to social media, or concern from healthy-weight children during changes at puberty). The relevance to children of all body weights was considered important to reduce stigma so that simply having a copy of the guidance would not signal that a family included a child living with overweight or obesity.

### Phase 3: Modified Delphi Study

#### Recruitment

In line with guidance on conducting Delphi studies [45] we aimed to recruit 20–30 participants representing different stakeholders and areas of expertise. Six core groups that should be represented were identified, with a target of recruiting a minimum of two people within each group: (1) parents of primary school aged children, (2) school nurses involved in delivery of the NCMP, (3) public health professionals working in children’s healthy weight, (4) other health professionals involved in the general care of children (e.g., GPs, health visitors, dieticians), (5) clinical child and adolescent psychologists, and (6) relevant academic researchers.

Recruitment was initially through the existing networks of the study authors, drawing on knowledge of published researchers specialising in this area and contributors to the PHE, now OHID, NCMP board. An open call for expressions of interest was also posted on Twitter (originating from the lead author’s account but retweeted by other authors to their networks). Twitter volunteers were screened from their Twitter/internet profile on the basis of (i) credibility (e.g., employing organisation, qualification, length or nature of experience), (ii) uniqueness of insight (e.g., international experience, representation of charity etc.), (iii) impact on the balance of expertise across the Delphi group.

#### Procedure

The modified Delphi process consisted of two rounds of review and response; a third was scheduled but consensus had been reached at the end of Round 2. Ahead of each round, participants were emailed documents to review, alongside a short video talking through the materials and outlining the aims of the round.

In Round 1, the documents for review included the draft guidance (Draft 1), a brief overview of the findings from Phase 1, and links to more detailed information for those wishing to give greater scrutiny (outline of approach sent to participants is provided in Additional File 4). Participants provided feedback via a Jisc online survey [46] with a primarily open response format seeking comment on each section and page of the guidance. For each page in turn, survey questions asked whether there was anything missing, whether anything should be removed, and for any other comments (e.g., changes in tone, adaptations etc.). A small number of closed questions were also included (e.g., whether case studies were helpful or not). In Round 2, participants could provide back using the survey, through a webinar (held before the deadline for completion of the Round 2 survey), or both. A short video was produced by the research team setting out the rationale for what was changed or kept the same and why, which was circulated with the updated guidance. The webinar was provided in order to allow participants a chance to discuss points of contention, giving an opportunity for people to explain their opposing views and a chance to arrive at consensus. Employees from the organisation it was intended would implement the guidance (PHE, now OHID) were also invited to listen to, and contribute to the Round 2 discussion. This was done to involve them in discussions that related to future pragmatic and feasibility decisions such as document length, formatting and use (*n* = 4).

After each phase, all comments were extracted and discussed by the two lead authors, FG and EG. A response to each comment was made and recorded; a “*You said, we did*” feedback document reporting the key points of feedback received from Round 1 alongside the changes subsequently made was included with the revised version of the guidance (Draft 2) during Round 2 (Additional File 5). Decisions on whether or not to implement a change were based on (i) frequency of similar comments across participants, (ii) background of respondents (e.g., for matters of opinion/preference, views of parents or those working most closely with parents and children were prioritised) and (iii) feasibility within the aims of the guidance (e.g., it was not considered feasible to add sections beyond the scope of communicating about weight). Areas of disagreement or wide variation in views after Round 1 (e.g., over the extent of the use of the word “weight” throughout the document) were discussed by all authors to agree an approach for compromise. A justification for the agreed text was then presented to the Delphi group for final confirmation.

## Results

Twenty-nine participants provided feedback to Round 1, and 26 to Round 2, with 11 (not including the authors) attending the webinar (Table 2). Most participants responding to demographic questions (*n* = 21, 81%) were white, two were Asian, one black, and two from a mixed ethnic background. The majority were in the 35–44 (54%) or 45–54 (31%) year old age ranges, and 65% had children themselves, mostly aged 18 and under.

**Table 2:** Participants at each stage of the Delphi process

Stakeholder group	N invited	N responding to Round 1	N responding to Round 2	Participated in Round 2 webinar^b^
Parents of primary school aged children	4 (2 Male, 2 Female)	3 (1 Male, 2 Female)	2 (1 Male, 1 Female)	-
School nurses	4 (4 F)	3 (3 F)	2 (F)	2 (F)
Local public health advisors/specialists	3 (2F,1 M)	2 (1 M, 1F)	2 (1 M, 1F)	4 (3F, 1 M)
GPs	3 (3F)	3 (3F)	3 (3F)	-
Dieticians^a^/other health professionals	1 (M)	0	1(M)	-
Clinical psychologists	5 (3F, 2 M)	4 (2F, 2 M)	4 (2F, 2 M)	2 (1F, 1 M)
Academic researchers	19 (16F, 3 M; 2 North America)	14 (12 F, 2 M; 2 North America)	11 (10, 1 M; 2 North America)	3 (F; 1 North America)
Eating disorder charity represented	2 (unknown)	1 (unknown)	1 (F)	1 (M)^c^
TOTAL	41	29	26	12

^a^we recruited a practicing paediatric dietician but they did not respond

^b^this is a subset of the previous column (i.e., all webinar attendees were considered to have responded to Round 2)

^c^different representatives of the same organisation contributed at different time points

### Development process

Delphi participants’ responses to Round 1 (Draft 1) provided detailed feedback on areas for improvement in terms of the tenor of the guidance overall (Additional File 5). In Round 2, responses to the revised guidance (Draft 2) were consistent and far less extensive than received in response to the first draft, suggesting only minor further changes were necessary. In response to closed questions to the participants, all respondents reported the changes to the guidance had made Draft 2 sufficiently positively framed, more readable (shorter and less complex language; although three requested further adaptations), and 22/26 agreed that the revised version was successful in reducing implied blame for parents.

Discussions during the webinar focussed on; (i) whether and where to refer to ‘weight’ specifically, (ii) better reflecting diversity and (iii) how to reduce parents’ feelings of blame. (i) Respondents were concerned that the guidance should primarily advise parents to have conversations around health, growth and energy rather than weight, and that although there was agreement that ‘weight’ should not be a banned word, there was agreement that the guidance should not appear to encourage more conversations about weight than necessary. (ii) It was noted that most images of children obtained through free-to-use photo libraries (e.g., [44]) present an idealised family life, for example with two parents of different genders in visibly affluent homes. To be more inclusive we were directed to find images including more socio-economic and ethnic diversity, to remove colloquial language, and to use a more diverse set of names, food and activities in case studies. (iii) A greater emphasis on external influences on the development of obesity was endorsed, to try and reduce parents’ feelings of self-blame.

A third version (Draft 3) was created in response to the Round 2 feedback. This was then piloted with school nurses, health screeners and parents who received feedback through the NCMP that their child was overweight or very overweight in the final term of the 2020/2021 academic year. The results of the pilot study are reported elsewhere [47]. Draft 3 is available in supplementary materials, and formed the basis for professionally formatted versions to be used in applied settings.

## Discussion

This paper reports the process of creating evidence-informed guidance, reflective of clinical expertise, to support parents to talk about weight with their children in a way that can support the child’s wellbeing. The guidance could be used as an adjunct to the process of weighing and measuring children as conducted through the NCMP, or at other points when parents may want or need to talk to their child about weight, for example following health consultations or children’s reactions to online content or discussion with peers. In line with what is described in the new MRC complex interventions guidance [48], we consider the guidance to be an ‘event within a system’ by making conversations that happen between parents and children about weight a more positive experience, in support of other interventions that may take place alongside this. It is intended that parents will feel more confident in how to handle such conversations and, as a result, less anxious of the impact on their child’s wellbeing of other steps they may take to improve children’s health (e.g., when making changes to the family’s diet or physical activity that may happen without the need to discuss weight). Importantly, it is intended that children will have more positive and supportive experiences in talking about their own weight with their parents or carers which may be an important part of reducing the increases in children’s weight dissatisfaction [16, 17].

The process of development reported here provides an example of an approach that could be implemented in other settings to translate multiple disparate sources of information into practical tools where existing guidance, or categorical evidence, is lacking [48]. The engagement of external stakeholders through the modified Delphi process in Phase 3 worked well in enabling us to engage and consult with a wide range of people, with sufficient structure (mapping each point of feedback to a specific aspect of the guidance) that it was usable. Participants reported that providing short video explanations to accompany each phase was particularly useful in helping them to understand the task, without feeling the scale of the task to be overwhelming. Providing a webinar allowed participants to elaborate on their responses, and an opportunity for people with different views to discuss how we might compromise; these discussions also provided the research team with a broader understanding of the reasons behind particular views which could be applicable to responding to other submitted responses. Nonetheless, the webinar may have been intimidating for those less used to expressing views, and notably no parent representatives attended. Time limitations also meant that only some of the points of contention were discussed further. While preliminary pilot work suggests that the guidance is considered useful and acceptable [47], it will be important to test the hypothesised mechanisms of effect as set out in the logic model before there can be greater certainty that this guidance has a meaningful impact.

The process of raising parental awareness of children’s weight status has been criticised due to the association between parent awareness and children’s weight concern, and the absence of strong reported benefits on their physical health [15, 49]. However, the case for providing the guidance for parents that we have reported here is relevant and important whether or not such systems exist; HCPs report the need for guidance for parents based on their one-to-one practice experience [47], and many parents who perceive their child to be overweight and are concerned at how to address this have not learned about their child's weight through national programmes. Further, as rates of children’s weight concern and weight loss attempts increase [17], parents of children of all weights may wish to seek advice on how to help their children to develop more positive self-perceptions and behaviours in relation to their body size and weight. Other work with children and young people, for example through work conducted with 630 young people by the Royal College of Pediatrics and Child Health (RCPCH) State of Child Health (Healthy Weight), corroborates the finding that when they are consulted, many children are interested in the opportunity to find out whether or not their weight is healthy through routine checks [49, 50].

### Strengths and limitations

The strengths of this report reflect the extent and breadth of academic and non-academic evidence and expertise that informed the process and the outcome of the programme of work. This incorporated diverse views from lay, clinical, public health and academic contributors through a systematic process in which decisions were documented and scrutinised through an iterative process of engagement in the creation of a resource. The success in engaging contributors—as well as the perceived importance of this work—was evident in the high retention rate in the modified Delphi study, and that we obtained a sufficient Delphi sample size to be likely to provide stable results [45].

Nonetheless, the quality of the output is limited by the evidence available to inform it. There is little research using prospective or experimental designs that can provide more specific feedback on the outcomes of discrete elements of the document content (i.e., specific types of communication). For example, there is no evidence to provide a definitive answer to parents on whether or not to talk to children about weight, and from what age. Similarly, as the guidance is generic and designed for application at scale, it does not allow for tailoring based on the characteristics of individual families; for example, the recommendations in the guidance may be differently effective if delivered in families with different typical parenting styles, personalities or skill levels, and if received by children with different experiences outside the conversations they have with their parents (e.g., those being bullied or teased because of their weight, and those more versus less concerned about their weight). While the ethnicity and gender among the Delphi panel was varied, the research team are all female, white and British. As discussed, consideration was given to representing families from diverse backgrounds within the guidance, but it has yet to be piloted with people from non-Western cultures and communities for whom English is not a first language.

### Future research

Specific research is needed to test the impact of this guidance on parental confidence and skills in relation to talking about weight, and if these are improved, whether this results in experiences that are more positive for children. This would include testing the proposed mechanisms of effect set out in our logic model, and those assumed within public health practice more generally (e.g., of the benefits of, and ethics around raising parental awareness of childhood obesity). In considering the contribution that improving parent communication about weight to children could have on the wider context, it would also be useful to explore what impact would result from circulating this guidance among all parents, not only those whose children are identified as being overweight; that is, whether the guidance collated here, whether in this or alternative formats, help to shift perceptions of blame and stigma in relation to children’s weight among the whole community.

Research would also be valuable to develop a better understanding of what changes for children who are identified as having overweight or obesity through the NCMP as a result of the feedback that is provided to parents (e.g., whether it results in more conversations, changes in self-perception, or changes in the home environment etc.). This would help us to understand the mechanisms and determinants of the observed associations between child and parent perceptions of overweight and subsequent development of weight concern [14], including whether and how these may be modifiable.

## Conclusions

This paper has used the process of evidence synthesis, and expert consensus, bringing together insight from across disciplines and groups to extend our knowledge of how to advise parents to talk to children about weight. It provides a single-source, evidence-based repository with the potential to influence public understanding and health professional practice. The process taken demonstrates that in this highly sensitive topic area, we were able to engage people with very different concerns and perspectives, bringing them together through a common desire to prioritise children’s wellbeing, to achieve broad consensus on what advice and tools are useful and appropriate to parents needing or wanting to talk to their child about their weight. The next step is for the guidance tool to be tested at scale and further developed for delivery through different media and formats. By conducting this work through a systematic and auditable process, mapping specific elements to their predicted effects, we have also provided a tool in which the contribution of elements and the sum of the parts are measurable, and provide a framework for enhancement.

### Supplementary Information

**Additional file 1.** Review of existing guidance and resources.

**Additional file 2.** Detail to support reported feedback from consultation with children.

**Additional file 3.** Information sent to Delphi Participants in Phase 1.

**Additional file 4.** Feedback provided to Delphi participants following Round 1 revisions to the guidance document.

**Additional file 5.** Guidance document in format agreed by Delphi Participants.

## Data Availability

All data generated or analysed during this study are included in this published article [and its supplementary information files].

## Abbreviations

HCP: Health Care Practitioner
NCMP: National Child Measurement Programme
OHID: Office for Health Improvement and Disparities
PHE: Public Health England
