# Supplementary material for: The systematic development of guidance for parents on talking to children of primary school age about weight
Source: BMC Public Health. 2023 Sep 4;23:1704. doi: 10.1186/s12889-023-16527-5 (PMC10476424; doi:10.1186/s12889-023-16527-5)
Supplement: Supplementary file 2 — Additional file 2. Detail to support reported feedback from consultation with children. [file 12889_2023_16527_MOESM2_ESM.docx]

**Additional File 2: Detail to support reported feedback from consultation with children**

# Outline of study methods

Ethical approval for the study was obtained from the Institutional Ethics committee (REACH reference number EP18/19 113). While it was intended that the interviews would take place in person, recruitment began in 2020 just as the Covid-19 pandemic began, so all interviews took place virtually using Teams and recruitment took place using online paid for adverts delivered through Facebook, school newsletters and University publicity.

Participants: Participants were parent and child dyads, eligible if the child was between 8 and 11 years old, without a diagnosed eating disorder. The parent was present throughout the interview but asked to be present to support the child in understanding and answering questions, and not to answer for them.

Procedure: Parents were emailed an information sheet about the project (a version for adults, and a version for children), and those interested in taking part asked to complete and return a consent form by email. Parents were also asked to complete an online questionnaire reporting on their child’s demographics (ethnicity, gender, height and weight), and parent educational level as an estimate of socio-economic status. Oral assent was obtained from the child at the start of the online session once the researcher had had the chance to talk them through the study and check their (and their parent’s) understanding. Families were informed they could withdraw from the study at any time during the interview, and up to two weeks following the interview (after which time transcribing and analysis may have taken place). Children were provided with a small active toy to thank them for taking part.

Interviews lasted around 15 minutes and were recorded either through a separate audio device, or through Teams, immediately downloaded and deleted from the stream. All recordings were then transcribed and anonymised, allocating a pseudonym to each participant.

Interview topic guide: After building rapport with the interviewee, the researcher shared her screen to show the children some illustrated cards containing simple, incomplete stories, and asked them to think about how the child in the story may be thinking and feeling (see Appendix). The stories depicted a child being weighed and measured, as takes place within the NCMP. The researcher used a series of prompts to ask the child to ‘fill in the gaps’ about different aspects of the process including; how it feels to be weighed, why they think adults find weight important, what children would expect or want to be told afterwards, and how they may expect or want parents/guardians to respond. At no point was the term obesity used, but instead when appropriate we referred to children ‘who are a bit heavier than other children’.

Analysis: A framework analysis was conducted by EG, by coding the content of each interview to extract key messages for the guidance development. Codes related to;

- How children feel about being weighed and measured
- Why we weigh children and inform parents, rather than children themselves
- Whether or not parents should tell their child about their weight status, and what children expect them to do with the information
- Whether children feel weight status is something that can change

Patterns in responses were grouped as themes, reported below.

# Summary Results

Eleven children (7 female, 4 male) were recruited by the time analysis was needed to progress the Delphi study; one child was of Asian ethnicity, and the remainder White British. The socio-economic background of the children’s families was high; all families had at least one parent in employment, and only one parent was job seeking; eight children were living in two-parent households; and eight children were living with at least one parent with a University degree, with the remainder having at least A level qualifications. A summary of key themes and findings is presented in Table 1.

**Table 1: Themes and quotes from children relating to the question framework**

| Theme | Summary | Indicative quotes |
| --- | --- | --- |
| Children are aware of weight stigma | Children believed that being overweight would be something to be embarrassed by, and that is a reason for bullying. | *Children would want to talk with their parents [about weight] but not really anybody else because they might be embarrassed*. (girl, aged 9)  *Because it’s their own personal, their own personal information about their body and they might feel embarrassed if anyone, anyone else had that information.* (boy, aged 9)  *For some people it might be quite hard [to talk about weight] because they might feel embarrassed*. (boy, aged 9)  *[If parents don’t discuss weight] they might think that their parents won’t like them if, if they’re not the right weight.* (boy, aged 9)  [Do you think it's hard for children to talk about weight*?] For some children that have like a very, that has a healthy weight, well, it would kind of be easy for them because they know they’re, they have a good weight and they don’t need to worry about, um, being a bit too heavy, unhealthy weight and some children that have unhealthy, um, that have an unhealthy weight will, will kind of worry, because they don’t want to get bullied for being, for having that unhealthy weight... sometimes they just don’t really want to talk about it, because it might, just because they don’t really want to, I guess.* (girl, aged 11) |
| Children are accepting of being weighed and measured | Children see these measurements as a way to check they are healthy - but appreciated that it is a private health matter that shouldn’t be discussed with other children.  However, some children suggested children would feel nervous about being weighed in case their weight ‘wasn’t right’. | *I would feel fine, because I know I’m just getting weighed and measured for my health, so, but some people just, um, they’re like a bit more nervous to see if they’re, they’ve got like a healthy weight or not.* (girl, aged 11)  *The children will want their parents to see, because like they’re, the chil- they’re, they’re the children’s parents, so, um, the child will want, uh, the parents to see that they’re growing properly and that they’re healthy and that the school nurse thinks they’re healthy* (boy, aged 9)  *It’s their own personal, their own personal information about their body and they might feel embarrassed if anyone, anyone else had that information*. (boy, aged 9)  *If all the children were shown their weight then they might compare them or they might say, I’m heavier than you, or, you’re heavier than me, or, and then it would be kind of like bullying.* (boy, aged 9)  *[children might feel] a bit nervous… Because they’re going to see how much they weigh and they might not like that* (girl, aged 9) |
| Children mostly understand the link between diet, physical activity and weight | Children understand that diet and physical activity affect weight and health. They believed a person’s weight could change. | *[Measurements are done] to see if they’re healthy… to check that they're growing properly*. (boy, aged 9)  *Exercise is the important thing for weight* (boy, aged 9)  [Can someone change their weight?] *Get more fit...Get him in more healthier activities… Watch Joe Wicks!...Eat healthier...More fruit and veg like me.* (boy, aged 11)  *[If they were overweight] they could do more exercise and eat less things with like sugar in it and like go on sort of like a diet.* (girl, aged 10)  *… it doesn’t matter how much you weigh or if you’re small or big, you’re still a human, but the important thing that matters is that you’re healthy. [*And would your weight matter for your health?*] it might be important in some, in some way, but in others not so much. So, it would matter when the doctor is checking that you’re growing properly* (boy, aged 9) |
| On balance, children thought they should be told about their weight. | Children were often initially unsure or ambivalent about whether they should be told their weight, but on reflection felt that children should be told, and that it was important for them to know if they are healthy or not.  Children could also see that parents may not talk about weight so as not to worry their child. | *They [parents] should tell [children their weight] so [we] know like what to do to get fitter.* (boy, aged 10)  *I think I’d kind of like to know my weight… I'm just curious* (boy, 9)  *If it’s a problem it needs to be talked about, but if it’s, um, if you’re normal that’s also great, because that’s great news, so I think that’s an important thing to discuss.* (girl, aged 11)  *If [children] were like overweight or something like that [parents] might [not tell them about their weight as they might] not want them to feel bad about themselves*. (girl, aged 10)  *They could either tell their children or they could just keep it a secret… depends on the results…. if they were either healthy or, um, could be a bit healthier.* (girl, aged 10) |
| Children’s expectations of what parents should do if a child is overweight | There was confidence among children that parents would help them to manage their weight and health if they were overweight. | *Your parents can give you information on how to improve your weight and how to, and what to do to, um, to build your weight up properly.* (boy, aged 9)  *[Parents should] talk to the child [*And what might they say*?] like can you, like, I know you love chocolate and all, but like you can stop eating that and maybe try some exercise or try a new sport...Only the ones that are being lazy.*(boy, aged 10)  *Well they might tell the children and if there is something wrong with their children then they might go to the doctor. They’ll deal with it really well-ly.* (girl, aged 9) |

Appendix 1:

Story cue cards for interview (Version 2, 06/12/2019)

This is Berrybrook Primary School. One day, during an English lesson, the school nurse came in to measure all the children in class 6A.

<*Picture of a school presented here for illustration>*

The children were asked to go out one by one to be measured in an empty room so no one else could see.

<*Picture of weighing scales presented here for illustration>*

**Q1: What do you think the children would be thinking or feeling when that happened?**

Prompts:

- Why do you think school nurses weigh children?

All the children get weighed, but the nurse doesn’t tell them what the scales say. Instead, the nurse tells the children that their parents will get a letter with the results.


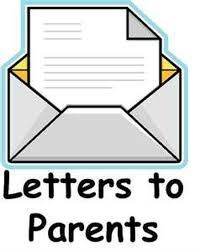


**Q2: What do you think about that?**

Prompts:

- What do you think the letter will say?
- Why do you think the nurse just sends the letter to parents, rather than letting the children know straight away?

**Q3: What do you think parents will do when they get the letter?**

Prompts:

- Do you think they will they tell their child what it says?
- Will they do anything else?

One week later, some of the children are talking about the letter that their parents got in the post about their weight. One of the children, Alex, has not been told about the letter by their parents.

<*Picture of a family eating together presented here for illustration>*

**Q4: What do you think Alex feels when their parents don’t talk about it?**

Prompts:

- Do you think it matters how much children weigh? Why/why not?
- Do you think anybody can do anything about a child’s weight? Why not/what could they do?
- How do you think we can tell if someone is healthy?

The letter that Alex’s parents got says that Alex is a bit too heavy for someone of their age and height.


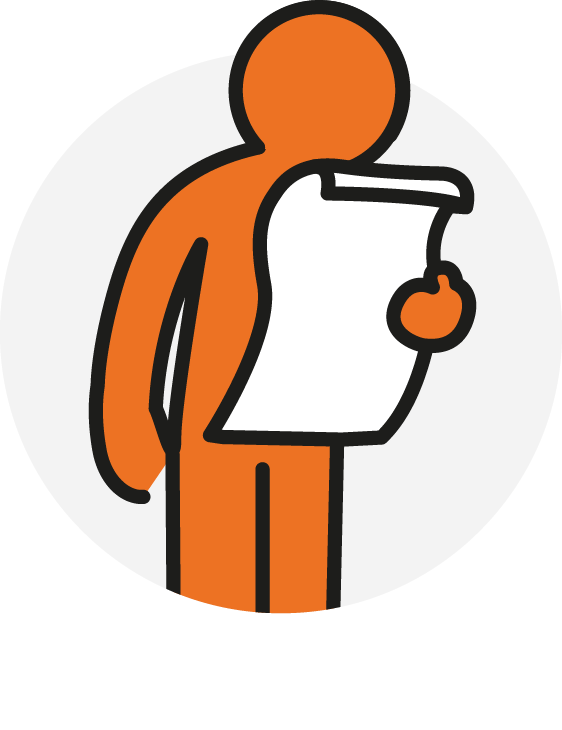


**Q5: Why do you think Alex’s parents don’t tell Alex?**

**Q6: Do you think Alex would want to know? Why/why not?**

Prompts:

- What do you think mums and dads should do?
- Do you think it is good for parents to talk with their child about weight? Why/why not? Is it important for all children or just some children (e.g. those who weigh more)?
- Do you think children would like to talk about their weight? What would they like to know?
- Is it hard or easy to talk about weight? Why?
