# Supplementary material for: The systematic development of guidance for parents on talking to children of primary school age about weight
Source: BMC Public Health. 2023 Sep 4;23:1704. doi: 10.1186/s12889-023-16527-5 (PMC10476424; doi:10.1186/s12889-023-16527-5)
Supplement: Supplementary file 3 — Additional file 3. Information sent to Delphi Participants in Phase 1. [file 12889_2023_16527_MOESM3_ESM.pdf]

Additional File 3: Information sent to Delphi Participants in Phase 1

Developing guidance for parents on talking to their child about their weight

Summary of three primary information sources underpinning the draft guidance

Public Health England

Core development group:

Fiona Gillison, University of Bath
Lis Grey, University of Bath
Angel Chater, University of Bedfordshire
Lou Atkinson, Aston University
Alison Gahagan, PHE
Anh Tran, PHE (rapid review)

University of Bath

Aston University Birmingham

University of Bedfordshire

Rapid review of studies promoting parent-child communication about sensitive health topics

What evidence is this review based on?

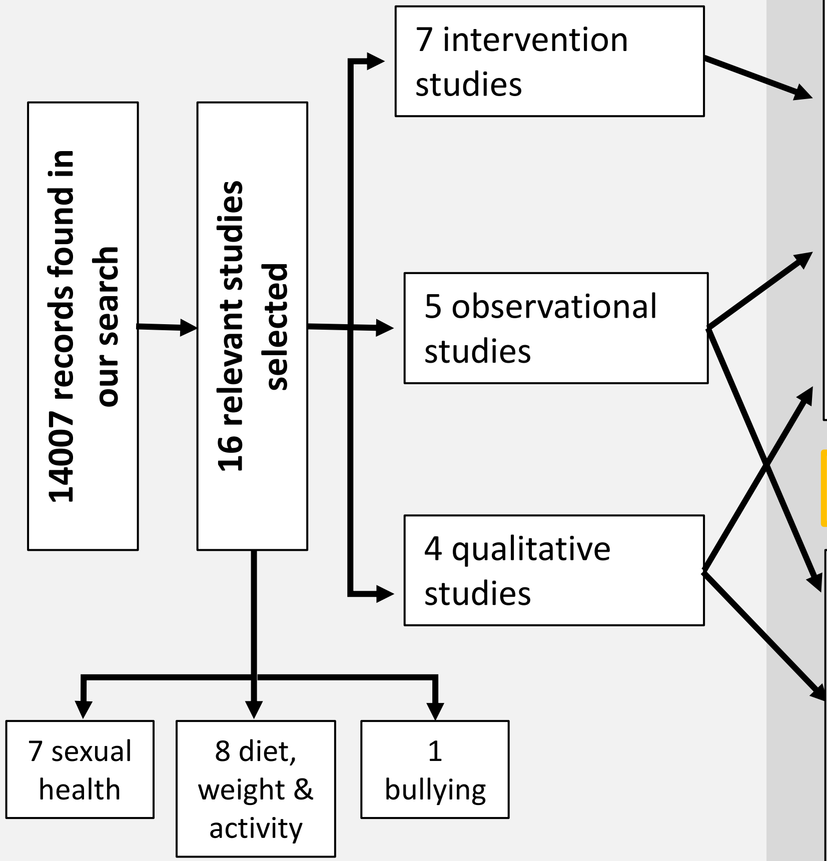

1) How can we encourage parents to initiate communication/communicate more?

- Educate parents on the impact they have on their child
- Educate parents on the potential benefits for children of talking about concerns and/or changing behaviour
- Consider a different approach for mothers vs fathers
- Repeated/ongoing exposure to the message can increase likelihood that conversations happen up to a point
- Improve confidence in parenting skills in general, and talking about the topic specifically
- No evidence that it helps to engage parents and children together

2) How do we advise them to communicate?

- Ensure the conversation is two-way; ask children questions (what they know and how they feel) and let them ask questions in return
- Include children in decision making
- Include children in deciding on what language to use
- Focus on growth and health, not size and weight
- Have more than one conversation – keep talking

How confident can we be in these findings?

Quality ratings:

- 3 studies rated as strong
- 12 studies rated as moderate
- 1 study rated weak

Relevance/questions unanswered:

- Not many studies on any one topic, so hard to make conclusions about consistency of findings
- No intervention study of the content of parent-child communication about weight/health behaviours (i.e., addressing question 2)
- No study tested the impact of parents communicating vs not communicating about weight on children's outcomes

Review of existing guidance in the field of weight communication with children

Key resources

14 resources included

- 6 authorship unclear
- 8 expert authorship

Three most closely addressing this task:

- *Talking to your child about their weight: your questions answered* (Weight Concern, Date not known)
- *Weigh in: Talking to your children about weight and health* (STOP Obesity Alliance and Alliance for a Healthier Generation, 2013)
- *Confident body, confident child* (La Trobe University, Australia, 2017)

Common content among all guidance:

- Acknowledging why parents don't want to talk about weight with children
- Information on healthy weight and consequences of overweight
- Suggestions for what to say in certain situations/response to children's questions

Specific advice to:

- Avoid teasing children about weight or using stigmatising terms
- Aim to increase children's self-esteem
- Don't avoid talking about weight
- Encourage children to talk about how they feel about their weight and ask questions
- Don't promote losing weight or dieting
- Take a whole family approach to healthy eating and activity; make sure all parents/caregivers are 'on the same page'
- Act as a role model – avoid criticising own or others' appearance

Gaps/weaknesses

- Little on dealing with social media, including;
  - How to address questions children raise about what they've seen
  - Dealing with social media bullying
- Tendency to focus on mothers and girls – unique content for fathers and sons may be needed
- Best available resources would need adapted for UK audience
- Even in the resources authored by experts and that cite research, this is limited by the lack of research out there

Guidance available but not included:

- For children with specific clinical conditions (e.g., diabetes, anorexia)
- Guidance for health professionals

Children's views: Outcomes from interviews

Qualitative interview study

11 participants (to date, study ongoing)

→ 9-11 years old, 4 boys/7 girls – all accompanied by mothers

→ recruited from across England via Facebook adverts to parents, school newsletters, University publicity

Interviews via Microsoft Teams

→ story cards shared on screen

→ questions on children's views of being weighed in school and talking with their parents about weight.

Additional sources consulted:

2 MSc students' projects (Aston and Coventry universities)**:** focus groups with 10-12 year olds (N=12). Aimed to explore children's views on the NCMP.

Blood & Grogan (2012): qualitative study with 12 children (10-11 years) exploring children's views on the NCMP.

In their own words…

On talking about weight with parents

They [parents] should tell [children their weight] so [we] know like what to do to get fitter. (boy, aged 10)

Children would want to talk with their parents [about weight] but not really anybody else because they might be embarrassed. (girl, aged 9)

If it's a problem it needs to be talked about, but if it's, um, if you're normal that's also great, because that's great news, so I think that's an important thing to discuss. (girl, aged 11)

Your parents can give you information on how to improve your weight and how to, and what to do to, um, to build your weight up properly. (boy, aged 9)

On not talking about weight

If all the children were shown their weight then they might compare them or they might say, I'm heavier than you, or, you're heavier than me, or, and then it would be kind of like bullying. (boy, aged 9)

If [children] were like overweight or something like that [parents] might not want them to feel bad about themselves. (girl, aged 10)

[If parents don't discuss weight] they might think that their parents won't like them if, if they're not the right weight. (boy, aged 9)

Key findings and themes

- **Children are accepting of being weighed and measured**

→ but they show sensitivity to weight status being a private health matter, and that sharing results with other children could lead to teasing/ embarrassment.

- **Children understand that diet and physical activity affect weight and health**

→ but would require guidance to change their behaviour safely.

- **Children thought that their parents should tell them their weight**

→ they felt that it was their information and that it was important to know if they were healthy.

→ while they reported that children might not feel good to be told they were an unhealthy weight, they were confident that their parents would help them in, and talk to them about, becoming healthier.

What next?

1. Feel free to read more about each of these three information sources, and see more of the data they draw on, in the appendices
2. Read through the draft guidance – we would like to hear your thoughts, based on:
   a. How well the guidance reflects the key, 'take-home' points listed here
   b. Your experience, whether as a parent yourself, or in a professional capacity
3. Complete the online survey available at: https://bathreg.onlinesurveys.ac.uk/delphi-part-1-developing-guidance-for-parents
4. If you wish to comment directly on the guidance document, please feel free to do so and email this to Lis at e.b.grey@bath.ac.uk

List of appendices:

- Summary of academic papers and existing guidance reviewed, with key points highlighted
- Draft report of our rapid review
- Interview schedule for child interviews
- Additional research outputs including:
  - Previous systematic evidence reviews
  - Individual research papers that contribute to specific points included

Please help us to keep up the momentum, by submitting your responses by Friday 8th January - thank you!
