# Supplementary material for: The systematic development of guidance for parents on talking to children of primary school age about weight
Source: BMC Public Health. 2023 Sep 4;23:1704. doi: 10.1186/s12889-023-16527-5 (PMC10476424; doi:10.1186/s12889-023-16527-5)
Supplement: Supplementary file 4 — Additional file 4. Feedback provided to Delphi participants following Round 1 revisions to the guidance document. [file 12889_2023_16527_MOESM4_ESM.docx]

**Additional File 4: Feedback provided to Delphi participants following Round 1 revisions to the guidance document**

**“You said, we did!”**

**Key points overall**

| You said…. | N* | We did…. |
| --- | --- | --- |
| Wording needs to be more direct, briefer, and less academic | >10 | Whole document revised to simplify wording |
| More positive framing needed, clearly setting out what parents can do, rather than presenting what they should avoid | >10 | We have reframed the guidance positively wherever feasible. On occasions where we feel that we need to suggest something to avoid, we have provided a suggestion of what to do instead. |
| Dealing with conflicting views among Delphi contributors (e.g., some consider it important to acknowledge that talking about weight is difficult, but others considered that stating this may exacerbate/create more concern) | 4 | We have tried to compromise through using double-sided statements, so the acknowledgement of the challenge is visible, but does not lead the agenda (i.e., it is presented with a positive statement, such as the benefits) |
| More is needed to reinforce how important parents are, in modelling language and weight-related behaviours, particularly in relation to their own weight | >10 | We have created a new section (now Section 2) called “Making changes together” to bring this together, presenting this ahead of more specific guidance on how and when to raise the topic of weight |
| Many respondents commented that it is important for parents to resolve the differences between parents/caregivers first, before they can start to create a more positive environment around talking about weight at home. | >10 | This is now a key part of our new Section 2 – “Making changes together” |
| More racial and physical ability diversity in photos is needed (both the people and the food) | 2 | We are grateful that this was flagged and have changed one case study to signal a BAME family and included more diversity in the photos used. Although the photos in this draft are not what will be used in the final guidance, they are included here to indicate that we will have images that reflect a diversity of ethnicities and abilities. |
| Check that we are not implicitly appearing to blame parents (e.g., inferring that parents are necessarily not providing a healthy diet already) – and that we actively include text that aims to minimise blame (e.g., signalling environmental contributors to rising obesity levels). | 2 | We have reviewed the text throughout to check we are not inferring parents are at fault/ providing an unhealthy environment and included some text about the challenges of keeping to a healthy weight given the obesogenic environment we live in (not in those words!) |
| While there was consistency about referring to health, growth (and the addition of function) rather than weight where possible, other contributors highlighted how to not talk about ‘weight’ may exacerbate stigma or fail to acknowledge children’s experience if they feel they can’t talk about it or that parents are avoiding the term. | >10 | As the document aims to directly address conversations about weight, we have not shied away from using this – however, in Section 3, we have added some text to directly reflect contributor’s concerns – i.e., to suggest that whether or not parents refer to weight rather than growth/health/function may depend on why and when they raise these issues, and whether their child refers to weight. **We will discuss this topic at the webinar** |
| Include an explanation of what is a ‘healthy weight’ | 3 | We feel that this is beyond the scope of this guidance as we want to keep the focus on talking about weight and there are many other resources that explain weight status. However, we have included a link to the NHS resources on this. |
| We need to make sure the guidance speaks to parents who may be talking about weight to much (e.g., calling their child fat, obsessing about their child’s weight, introducing dieting, weight shaming) as well as those unsure of how to talk about it. | 2 | We have added some content on this in Section 4 (now called “helping your child to feel good about their body”) |

**some may be the same person, but answering different questions*

**Front page**

| You said…. | N* | We did…. |
| --- | --- | --- |
| Change the title to include ‘healthy weight’ | 3 | We have decided not to change the title, partly to keep it short but also because we are trying to help parents talk about both healthy and unhealthy body weights with their child. |
| Use 'parents and caregivers', not just 'parents' - throughout document | 2 | On the front page, we have added ‘and caregivers’ to all instances of ‘parents’. We have also added a not here to explain that in the rest of the document, to keep it short, we use ‘parents’ to cover both parents and other primary caregivers. |
| There was some confusion over whether this guidance was only for parents of children with higher weights or for all parents | 2 | We have added a sentence to the first paragraph to clarify that the guidance is for parents and caregivers of children of all weights, and tried to make this clear at other key points. |

**Section 1**

| You said…. | N* | We did…. |
| --- | --- | --- |
| Provide examples of eating disorders in the box at the bottom of the page, as some readers may think this only refers to children who are underweight | 2 | Rather than listing eating disorders, which may not be well understood, we have rephrased the text to 'If you are worried that your child is not eating normally'. |
| Add normative info and emphasise that being overweight is not something to be ashamed about | 1 | Added that weight is not something to be ashamed about. We will discuss why we have chosen not to include normative information at the webinar. |

**Sections 2 and 3**

| You said…. | N* | We did…. |
| --- | --- | --- |
| *We have now combined Sections 2 and 3 (to form the new Section 3), as there was overlap between both of these and the new Section 2 – “Making changes together*” | | |
| You felt that examples are needed within this section, both of when are good times to have a conversation, as well as examples of wording | 8 | We have now integrated examples of both settings and wording that parents could use within this section |
| Some respondents advised caution (and asked for clarity) about statements relating to involving young children in decision making, and using their language choices, which may not be well informed. Similarly, a few respondents pointed out that children like to have boundaries, and that it is not always appropriate to defer to their preferences. | 8 | A more specific statement has now been included reflecting how and when children could be included in decision making. We hope the new terminology is clearer and more appropriate to when it is/isn’t appropriate to give children more choice. |
| Rather than asking about weight, ask about 'people being all shapes and sizes' | 2 | We have revised the whole document to make sure that we need to use the term ‘weight’ where we do so, and that we are clear when alternatives (such as this suggestion) may be more appropriate. |
| Include advice on how to challenge children on their own language (including towards their own parents) | 3 | We have included a statement on responding to children when they use ‘unkind’ terms both in Section 3 and in the scenarios. |
| Take care not to dismiss the links we all make between weight and looks (i.e., don't pretend that children’s experience is different to what it is) | 1 | We have included more discussion in Sections 2 and 3 about when and how to respond to different situations – so hope this is now clearer on not dismissing children’s experience. |

**Section 4**

| You said…. | N* | We did…. |
| --- | --- | --- |
| Add that although parents may be overweight themselves and have their own concerns, they can still make a positive difference to their child | 1 | We have now included this in Section 2 |
| Move guidance for parents about involving children, and managing their own concerns about weight to earlier in the guidance | 5 | We have introduced a new section this, in Section 2 |

**Narratives**

| You said…. | N* | We did…. |
| --- | --- | --- |
| Provide children’s ages | 3 | The children’s ages or school years have been added to the narratives. |
| Include with the narratives information about what the stories are based on (rather than front page) | 1 | As the narratives are already long, we feel it is better to keep this information in one place. However, in response to this and other comments (about what is needed first), it is now at the end of the guidance. |
| Re-order the narratives to better fit the points being made in the guidance | 2 | We have changed the order in the newly formatted guidance |
| The narratives are very long. | >10 | We appreciate that the narratives are long – they were written to include specific key points (in line with communication theory) so it is hard to cut them down without cutting out aspects that are designed to fulfil a certain function. We have amended these as per requests (i.e., for greater acceptability), but propose that rather than cutting length, we consider ultimately providing them in another format (e.g., audio or video). |
| Emphasise the healthy behaviours and the benefits of these over (and regardless of) losing weight | 3 | We have rephrased the narratives to emphasise becoming healthier in general. |
| A number of people suggested the removal of some phrasing/quotes – often those in more challenging language. |  | The narratives were designed to include the phrasing that families we had interviewed previously had used (e.g., being a ‘big kid’, or ‘chubby’). In places we have retained this wording for that reason (ensuring it is in quotation marks if so), but where nothing is lost by removing these quotes, we have done so. |

**Scenarios (below are the suggestions of new scenarios, that we haven’t included – with the reason why or how we have otherwise represented these in the materials)**

| Suggested scenarios | Reason why not included |
| --- | --- |
| Child not interested in/dislikes physical activity | Not included as it is more about changing a child’s behaviour rather than talking about weight or addressing concerns about weight |
| Child is upset that their clothes are for an age range higher/lower than their actual age | Not included, but we have included this in one of the examples in the section on “when to raise weight’. |
| Someone in the family/A family friend is diagnosed with diabetes/high blood pressure and your child overhears an adults saying that the diabetes/HBP was because of the person's weight | Not included due to space, less closely aligned to a child thinking about their own weight than the others included. |
| Disagreement between parents on how to approach the topic of weight with children and/or parents have different weights and activity levels | This is very important, but as a result of the revision process has now been made more prominent in Section 2 |
| Child says they don't care about their weight | This is challenging, and the best advice to give may depend on both the child and the parent. We think the main points in talking to children about weight that are already included in the guidance would be relevant here anyway. |
| Choosing food in a canteen | Not included as this is less likely to be a common scenario for children of this age to be doing unsupervised. |
| Parent disagrees with diagnosis/NCMP letter that child is overweight | This is already covered in one of the narratives. |
| Child makes negative comment about their own weight | This is included among the examples in the main sections. |
